# Experimental and simulation data of hole bearing type connections

**DOI:** 10.1016/j.dib.2024.110127

**Published:** 2024-02-01

**Authors:** Patrick Studer, Andreas Taras

**Affiliations:** ETH Zurich, D-BAUG, Institute of Structural Engineering, Stefano-Franscini-Platz 5, 8093 Zurich, Switzerland

**Keywords:** Bearing type connections, Experimental investigations, Finite element simulation, DIC measurements

## Abstract

This paper presents a comprehensive dataset comprising experimental test data and numerical simulations of hole bearing tests involving 32 single-bolt, 20 two-bolt, and 20 four-bolt specimens. The dataset encompasses load-deformation curves obtained from experimental tests and displacement data acquired via the Digital Image Correlation (DIC) system, which covers specific regions of the specimens. Additionally, the dataset incorporates force-deformation curves derived from corresponding numerical simulations.

The numerical simulation procedure is outlined, involving a simplified model employing solid elements for the specimen and rigid shell elements for the bolts. A “hard-contact” is employed to define the normal behavior of surface-to-surface contact between the specimen and the bolts. Material behavior modeling utilizes true stress–strain curves obtained from experimental tensile tests, encompassing both material properties extracted from these tests and the ensuing input parameters for numerical simulations.

Furthermore, the DIC-system measurements provide data on displacements and strain distributions across various regions of the specimens. These strain measurements are meticulously evaluated and presented.

The validation of the numerical simulations against experimental results substantiates the robustness of the numerical methodology, instilling confidence in its application for simulating bearing-type connections. Moreover, this dataset serves as a valuable resource for comparative analysis, enhancing the comprehension of these connections, and providing reference points for further numerical simulations.

Specifications Table

**Table:** 

Subject	Engineering
Specific subject area	Civil and Structural Engineering
Data format	Raw, Analyzed
Type of data	Tables, Abaqus input files
Data collection	Experimental test data was obtained from hole bearing tests. The tests were conducted with a Schenck 1600 kN universal testing machine. A linear variable differential transformer (*LVDT*) was installed on the back side of the specimen to measure the relative displacement between the specimen and the test fixture. The displacement field on the surface of the specimens was tracked quasi-continuously with stereo digital image correlation. The images were recorded using two monochrome cameras (type FLIR Grasshopper3 12.3 MP, 14.13 × 10.35 mm sensor) positioned at a distance of 700 mm (one and two bolts specimens) respectively 900 mm (four bolts specimens) to the specimen's surface. Related numerical simulations with the commercial software Abaqus were performed and the force-deformation behaviour evaluated.
Data source location	ETH Zurich, D-BAUG, Institute of Structural Engineering, Stefano-Franscini-Platz 5, 8093, Zurich, Switzerland
Data accessibility	Repository name: ETH Zürich Research Collection Data identification number: https://doi.org/10.3929/ethz-b-000636803 Direct URL to data: https://www.research-collection.ethz.ch/handle/20.500.11850/636803
Related research article	[1] P. Studer and A. Taras, “Influence of strain-hardening on the load-carrying behaviour of bearing type bolted connections,” Journal of Constructional Steel Research, vol. 191, p. 107185, 2022.

## 1 Value of the Data

• Integration of load-deformation data, DIC measurements, and validated numerical simulations yields a comprehensive dataset for hole-bearing type connections.
• The DIC measurements serve as a validation resource for numerical simulations.
• Researchers can leverage this dataset for comparative analysis and to augment existing datasets on bearing-type connections.
• Force-displacement data from hole-bearing tests can serve as benchmarks for designers' finite element models, ensuring accurate representation of mechanical responses in these connections.
• The presented numerical simulation approach and techniques offer valuable tools for designers in the realm of connection design.

## 2 Data Description

The csv-files in the folder “*01_Force_Displacement”* named *“SB_force_displacement_EXP”, “TB_force_displacement_EXP”* and *“FB_force_displacement_EXP”* present the data of the experimental tests for specimens with one bolt (SB), two bolts (TB) and four bolts (FB) respectively. The data is arranged column wise, where always two columns belong to each other, representing the deformation (*u [mm]*) and the force (*F [kN]*) for the corresponding specimen.

The data from the numerical simulations can be found in folder “*01_Force_Displacement”* as csv-files named *“SB_force_displacement_SIM”, “TB_force_displacement_SIM”* and *“FB_force_displacement_SIM”* for specimens with one bolt (SB), two bolts (TB) and four bolts (FB) respectively. The data is arranged column wise, where always two columns belong to each other, representing the deformation (*u [mm]*) and the force (*F [kN]*) for the corresponding specimen.

The tensile properties of the investigated steel grades, in the form of engineering stress-strain data, can be found in folder *“02_Material”* as csv-file *“Material_data_EXP”.* Where two tensile tests per steel grade are listed. In the same folder, the material data used for the numerical simulations can be found as csv-file named *“Material_data_SIM”* where pairs of true stress *σ_true_* and true plastic strain *ε_true,pl_* are arranged column wise for the four steel grades.

**Fig. 1:**
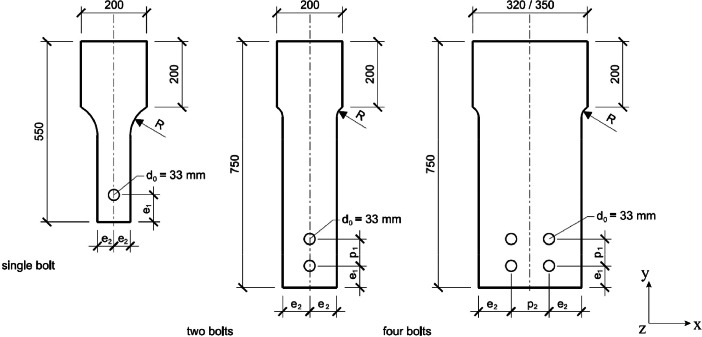
Specimens with one, two and four bolts including designation of the dimensions and the coordinate system.

Strains extracted from the DIC measurements can be found for each specimen in a respective Excel-file, which are located in folder *“03_DIC_Data”*, where three subfolders distinguish the files by the number of bolts (*SB*: single bolt, *TB*: two bolts and *FB*: four bolts). The Excel-files contain different sheets, which differentiates between the obtained data in the net-section(s) and below the bolt(s). The data is arranged so that each row corresponds to a certain force level. 50 points along the width of the specimens are extracted and for each point the coordinates (X, Y and Z), the deformations (U, V and W) as well as the strains ε_yy_ and ε_xx_ are extracted (see Fig. 1 for the coordinate system). The data for each point is arranged column-wise: X_1_, Y_1_, Z_1_, U_1_, V_1_, W_1_, ε_yy,1_, ε_xx,1_, X_2_, Y_2_, Z_2_, U_2_, V_2_, W_2_, ε_yy,2_, ε_xx,2_ and so on. Missing columns means that either no correlation could be found for this point, or the point is located inside the hole or outside the specimen.

In the folder *“04_Abaqus_INPUTS”* the input-files for the Abaqus simulations can be found. The are named as follows: Hole_Bearing_*a_b_c,* where *a* stands for the type of connection (SB: single bolt, TB: two bolts or FB: four bolts), *b* stands for the steel grade, followed by *c,* which are numbers representing the dimensions of the specimens according to Table 1.

**Table 1:** Nominal dimension of the specimens.

Specimen name		e_1_ [mm]	e_2_ [mm]	p_1_ [mm]	p_2_ [mm]	R [mm]	t [mm]
Single bolt	SB_10_15	33.0	49.5	-	-	100	7.5
	SB_15_15	49.5	49.5	-	-	100	
	SB_20_15	66.0	49.5	-	-	100	
	SB_30_15	99.0	49.5	-	-	100	
	SB_25_20	82.5	66.0	-	-	68	
	SB_25_25	82.2	82.5	-	-	68	
	SB_30_20	99.0	66.0	-	-	35	
	SB_30_25	99.0	82.5	-	-	35	
Two bolts	TB_15_25	49.5	82.5	82.5	-	35	
	TB_20_25	66.0	82.5	82.5	-	35	
	TB_30_25	99.0	82.5	82.5	-	35	
	TB_15_35	49.5	82.5	115.5	-	35	
	TB_30_35	99.0	82.5	115.5	-	35	
Four bolts	FB_15_25_25	49.5	99.0	82.5	82.5	40	
	FB_20_25_25	66.0	99.0	82.5	82.5	40	
	FB_30_25_25	99.0	99.0	82.5	82.5	40	
	FB_15_25_35	49.5	99.0	82.5	115.5	36	
	FB_30_25_35	99.0	99.0	82.5	115.5	36	

## 3 Experimental Design, Materials and Methods

### 3.1 Specimens and test-setup

In the experimental study presented and discussed in this paper, eight different specimen geometries with one bolt and varying edge distances e_1_ and e_2_ were tested. Additionally, specimens with multiple bolts and ten different geometries with varying edge distance e_1_ and bolt distances p_1_ and p_2_ were tested. All specimens were made from four different types of steel, resulting in a total of 32 single bolt and 40 multi-bolt connections. They were originally water jet cut from the parent plate, and in a second step the exact contour in the area of the taper as well as the holes were milled, using a CNC milling machine. The specimens with one, two and four bolts can be seen in Fig. 1 and the nominal dimensions can be found in Table 1.

The hole bearing experiments were conducted using a Schenck testing machine (F_max_ = 1600 kN). Whereby the quasi-static tensile load was applied to the specimens by the top grip displacement controlled with a constant rate of 0.025 mm/s. To facilitate the measurement of the deformation field surrounding the bolt hole using Digital Image Correlation (DIC), an asymmetric test fixture was devised. This fixture was engineered to enable a comprehensive view of plate deformations while minimizing any plate bending, thereby emulating a symmetric loading configuration akin to real-world applications, such as a plate element positioned between two splice plates.

**Fig. 2:**
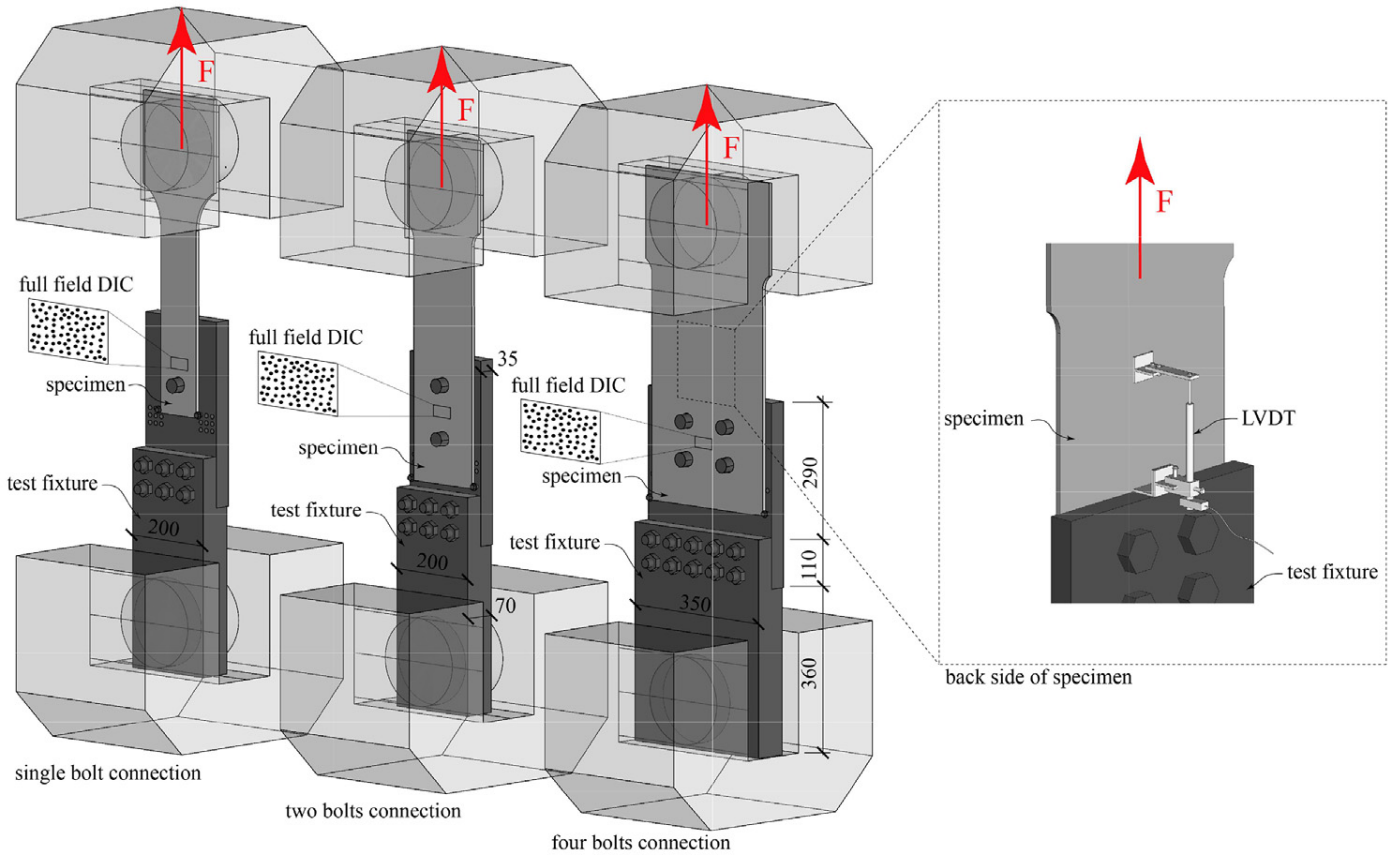
Test-setup with measuring instrumentation (dimensions in [mm]).

The test fixture comprised two steel plates, measuring 35 mm and 70 mm in thickness, respectively. These plates were affixed together using either six (in the one- and two-bolt test setup) or ten (in the four-bolt test setup) M20 (12.9) bolts. The upper plate was equipped with threads for M36 (12.9) bolts, specifically modified to 30 mm in the front area, spanning a length of 20 mm. A washer and nut were employed to secure the bolt on the rear side. Additionally, two M10 screws were utilized to prevent excessive movement of the lower part of the specimen out of the plane, thereby mitigating residual bending deformations. Fig. 2. provides a schematic representation of the test setup.

### 3.2 Measurement instrumentation

In conducting the hole bearing tests, the test rig's integrated load cell and displacement transducer were utilized to record the applied load magnitude and the overall elongation of the specimen, respectively. A Linear Variable Differential Transformer (*LVDT*) was positioned on the rear surface of the specimen, as depicted in Fig. 2c, to precisely gauge the relative displacement between the specimen and the test fixture. The data collection during the experiments was facilitated using the commercially available *catman* software by manufacturer HBK.

**Fig. 3:**
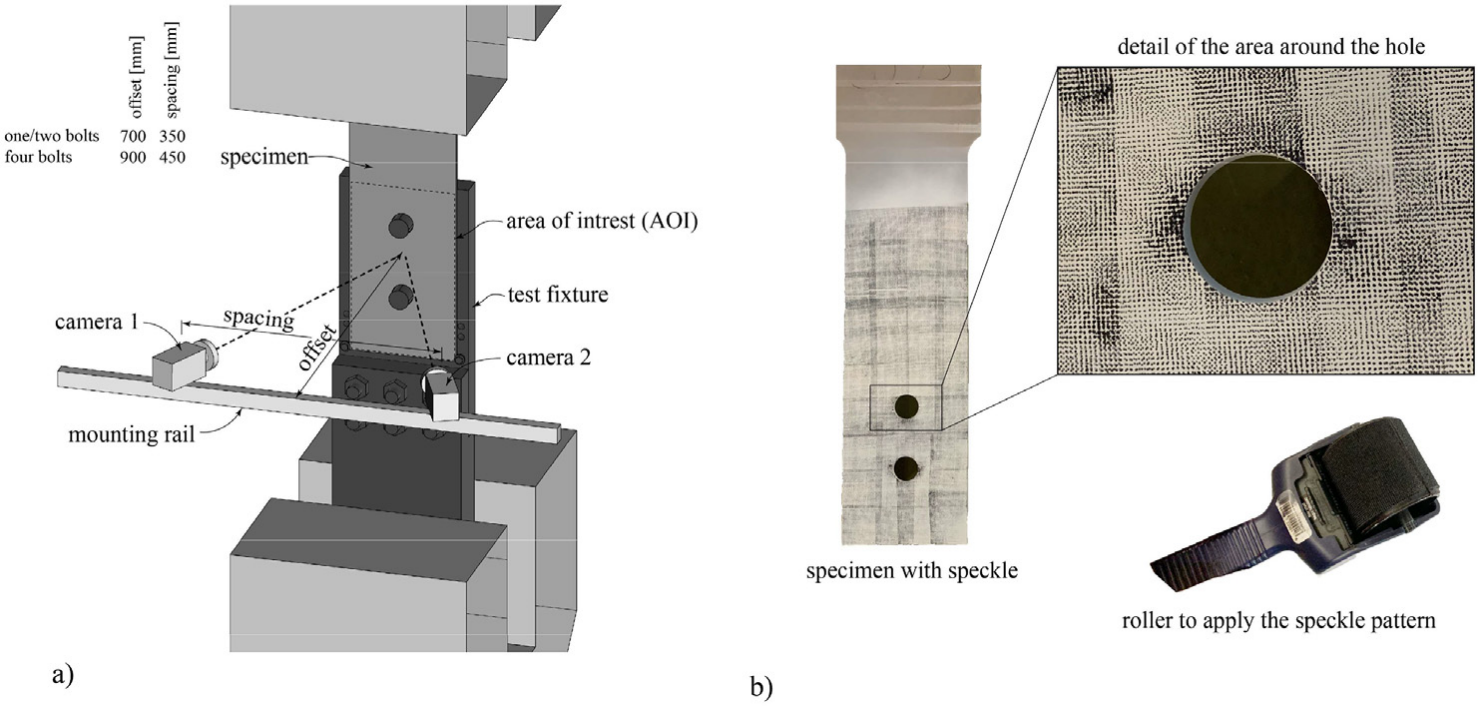
DIC measuring setup: a) schematic illustration and b) specimen with speckle pattern.

In addition to the *LVDT* measurements, the surface displacement field of the specimen was continuously monitored using stereo digital image correlation, ensuring quasi-continuous tracking. The speckle pattern employed consisted of black circular speckles measuring approximately 0.33 mm in diameter. This imaging process involved the use of two monochrome cameras of FLIR Grasshopper3 12.3 MP, each equipped with a sensor measuring 14.13 × 10.35 mm. Fig. 3a details the cameras' offset and spacing pertinent to different test setups. The specific configurations resulted in a stereo angle of 28°. Lenses featuring a focal length of 24 mm from Schneider Kreuznach were employed, providing a average scale of 0.10 mm per pixel.

A speckle pattern of randomly distributed black circular dots with a diameter of around 0.33 mm – which corresponds to approximately 3 pixels (should be in the range between 2 and 5 pixel according to [2]) – was applied onto a base layer of white paint to guarantee good contrast (see Fig. 3b).

The correlation was carried out with the commercial software VIC-3D (Correlated Solutions Inc. [3]) using different subset and step sizes according to Table 2. Further, a constant strain filter size of 15 was applied. For the calculation of the strains, the *Lagrange* tensor type was used.

**Table 2:** Subset size and step size for the different specimens with one bolt.

Specimen	Material	Subset size	Step size	Specimen	Material	Subset size	Step size
SB_10_15	S355J2N	25	5	SB_25_20	S355J2N	25	5
	S355M	25	5		S355M	25	5
	S460M	25	5		S460M	25	5
	S355M_SF	25	5		S355M_SF	25	5
SB_15_15	S355J2N	25	5	SB_25_25	S355J2N	25	5
	S355M	25	5		S355M	25	5
	S460M	25	5		S460M	25	5
	S355M_SF	25	5		S355M_SF	25	5
SB_20_15	S355J2N	25	5	SB_30_20	S355J2N	25	5
	S355M	25	5		S355M	25	5
	S460M	25	5		S460M	25	5
	S355M_SF	25	5		S355M_SF	25	5
SB_30_15	S355J2N	25	5	SB_30_25	S355J2N	25	5
	S355M	25	5		S355M	25	5
	S460M	25	5		S460M	25	5
	S355M_SF	25	5		S355M_SF	25	5
TB_15_25	S355J2N	41	7	FB_15_25_25	S355J2N	41	7
	S355M	41	7		S355M	37	7
	S460M	35	7		S460M	35	7
	S355M_SF	41	7		S355M_SF	35	7
TB_20_25	S355J2N	35	7	FB_20_25_25	S355J2N	31	7
	S355M	35	7		S355M	35	7
	S460M	35	7		S460M	27	7
	S355M_SF	31	7		S355M_SF	33	7
TB_30_25	S355J2N	31	7	FB_30_25_25	S355J2N	31	7
	S355M	37	7		S355M	33	7
	S460M	27	7		S460M	31	7
	S355M_SF	25	7		S355M_SF	37	7
TB_15_35	S355J2N	39	7	FB_15_25_35	S355J2N	35	7
	S355M	41	7		S355M	35	7
	S460M	39	7		S460M	41	7
	S355M_SF	35	7		S355M_SF	33	7
TB_30_35	S355J2N	27	7	FB_30_25_35	S355J2N	31	7
	S355M	33	7		S355M	27	7
	S460M	41	7		S460M	33	7
	S355M_SF	39	7		S355M_SF	29	7

### 3.3 Materials

#### 3.3.1 Tensile properties

Coupon tests were performed to ascertain the tensile characteristics of the four distinct steel grades under examination within this study. These tests, along with their subsequent evaluation, were executed in compliance with the guidelines outlined in *EN ISO 6892-1* [4]. This was conducted under the purview of our collaborative project partner, *voestalpine Grobblech GmbH*, as part of their internal quality control protocols. The yield strength of the material was determined based on the 0.2% proof stress criterion.

**Fig. 4:**
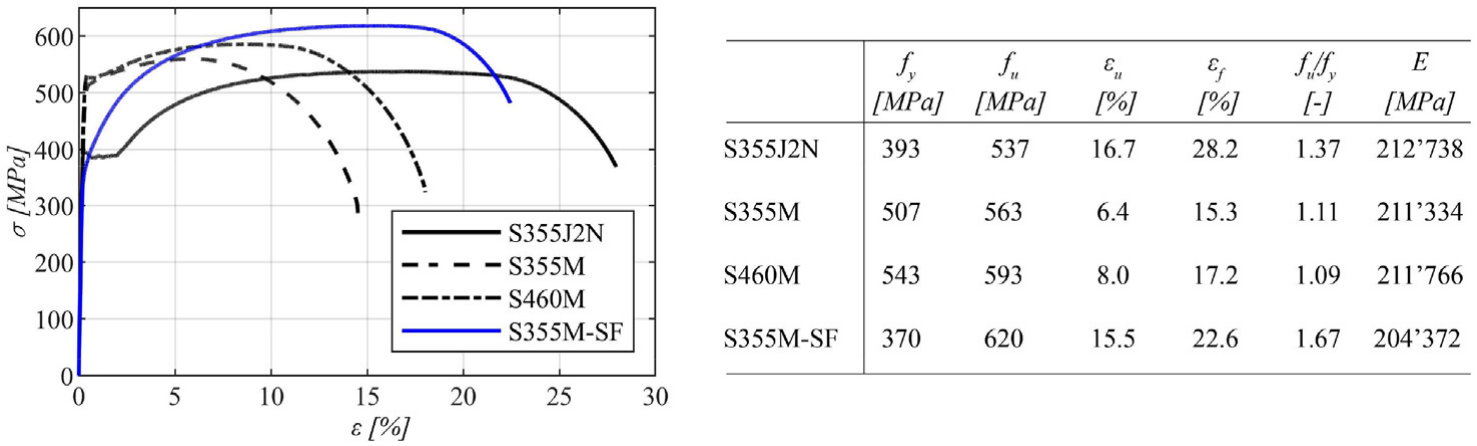
Tensile properties of the four different steel grades.

Two tests were undertaken in the longitudinal direction, and for analytical purposes, the average value derived from these two tests was considered. The collective outcomes of these tests are synthesized and visualized in Fig. 4.

#### 3.3.2 Numerical simulations

Numerical simulations of the hole bearing tests were carried out. The goal of the numerical simulations is to match the load-deformation curve of the experimental tests in the first part of the test until the maximum force is reached in an accurate way.

#### 3.3.3 FE model simplification, mesh and boundary conditions

**Fig. 5:**
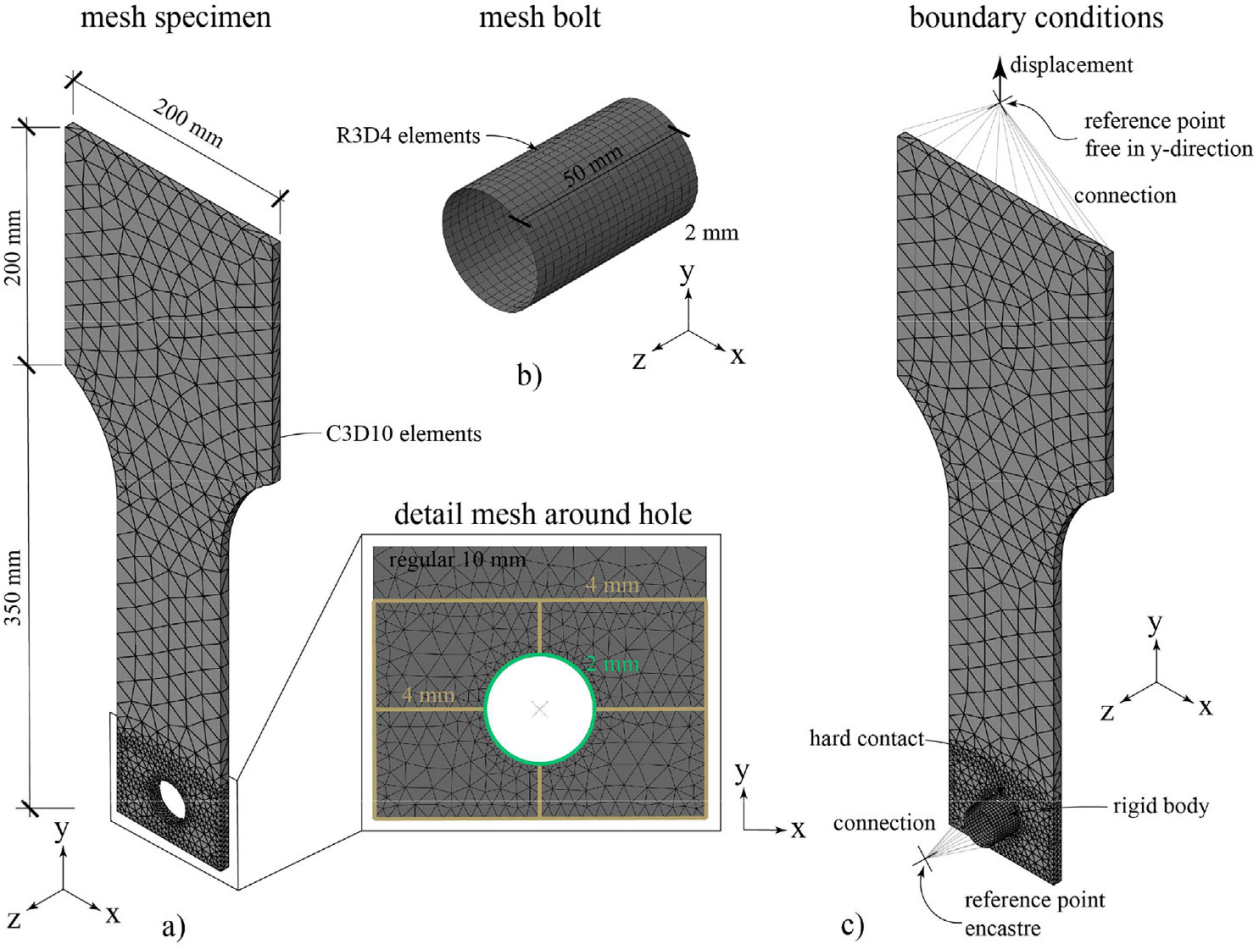
Numerical simulation a) mesh of the specimen, b) mesh of the bolt and c) boundary conditions.

The general FE analysis software ABAQUS [5] (version 2021) was used for the numerical simulation. The numerical model was simplified as can be seen in Fig. 5, where this is shown for specimens with one bolt but also applies to the specimens with two and four bolts. The standard implicit solver was used for the static analysis.

As the failure always occurs in the test specimen and not in the bolt, the bolt is simulated as a rigid shell element and meshed using R3D4 elements (see Fig. 5b), which are rigid three-dimensional elements with four nodes. The approximated mesh-size for the bolt is 2 mm and the length of the rigid bolt is 50 mm. For the specimen, solid elements were used and meshed by C3D10 elements, which are ten-node tetrahedral elements. A regular mesh size of 10 mm was used, which is refined around the hole to 2 mm and to 4 mm along the edges in the vicinity of the hole (see Fig. 5a). The input-files are created using parameterized input in commercial software MATLAB [6].

As can be seen in Fig. 5c, a rigid body condition is applied to the bolt and to simulate the gripping of the testing machine, the upper part of the test specimen is only free to move in y-direction. The static loading process used in the experiments is simulated through a displacement-controlled loading in y-direction applied to the upper part of the test specimen through a reference point which is connected to the specimen. The interaction between the specimen and bolt is simulated by a surface-to-surface contact, whereby a “hard contact” is defined as the normal behavior of this surface-to-surface contact. The master surface and the slave surface are the surface of the bolt shank and surface of the bolt hole respectively.

#### 3.3.4 Material modelling

For the numerical simulations, elastic and plastic material properties were defined. A Young's modulus, as depicted in Fig. 4, was employed for the four distinct steel grades, while a Poisson's ratio (ν) of 0.30 was established as a constant.

To specify the plastic properties within the ABAQUS software [5] (version 2021) pairs of true stress and plastic strain data were necessary inputs. These data pairs were derived from the engineering stress-strain curve, illustrated in Fig. 4. Accordingly, the procedural methodology proposed by *Hollomon* [7] was adopted. This method is frequently utilized to extrapolate true stress values post the onset of necking (as seen in other references such as [8]). The parameters characterizing the Hollomon equation for the four diverse steel grades are detailed in Table 3.

**Table 3:** Used parameters of the *Hollomon* equation.

*Hollomon* equation	$\sigma_{true}=K\cdot \varepsilon_{true}^{n}$	
Steel grade	*K*	*n*
S355J2N	883.86	0.1836
S355M	724.74	0.0704
S460M	771.03	0.0801
S355M-*slimfit*	988.81	0.1675

The true stress can then be obtained by the following equation.

(1) $$\sigma_{true}=\sigma_{eng}\left(1+\varepsilon_{eng}\right)$$

where $\sigma_{eng}$ and $\epsilon_{eng}$ are the engineering stress and strain, respectively. The true plastic strain can be determined using the following equation.

(2) $$\varepsilon_{true,pl}=\ln\left(1+\varepsilon_{eng}\right)-\frac{\sigma_{true}}{E}$$

**Fig. 6:**
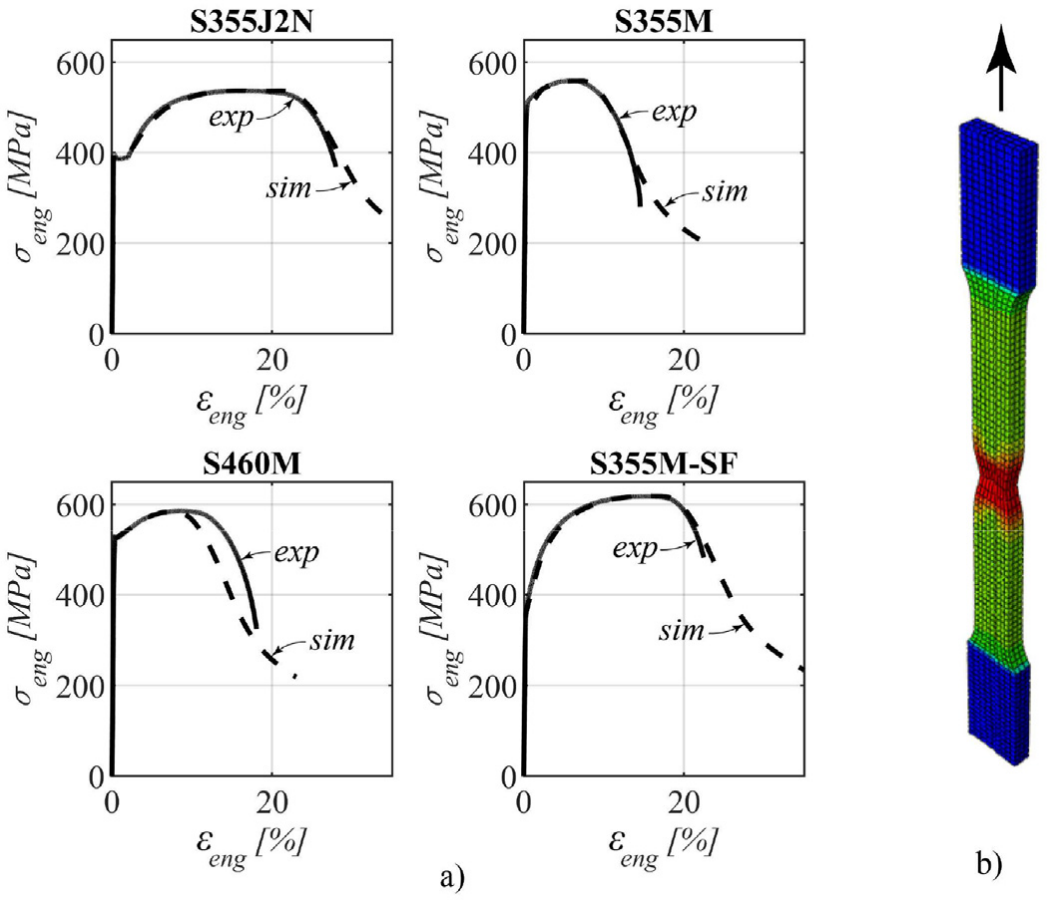
a) Comparison between experimental and simulated stress–strain curves and b) numerical model for coupon tests.

The numerical simulations of the standard tensile test were performed in order to obtain a stable numerical model that corresponds to the tensile test results. The coupon tests (see Fig. 6b) were simulated and a good agreement between experimental and numerical load–displacement curves could be obtained, as can be seen in Fig. 6a. Further, no damage model was used.

#### 3.3.5 Finite element model validation

Validation of the finite element model entails a comprehensive comparison of the load-deformation response with that derived from experimental tests. This involves a comparison of the maximum reached forces as well as an assessment of the force differentials throughout the test duration, up to the point corresponding to the maximum force in the experimental tests.

#### 3.3.6 Load–deformation behavior

This section presents the load-deformation characteristics of the specimens. Specifically, it involves plotting the load against the deformation measured with the *LVDT* during the experimental tests. To establish a common reference point, the deformation is referenced to the displacement at a load of 2 kN, ensuring that there is already an initial contact between the bolt(s) and the specimen. Furthermore, the load-deformation curves from the numerical simulations are overlaid as dashed lines, following the same reference procedure with the zero point of deformations set at a force of 2 kN.

**Fig. 7:**
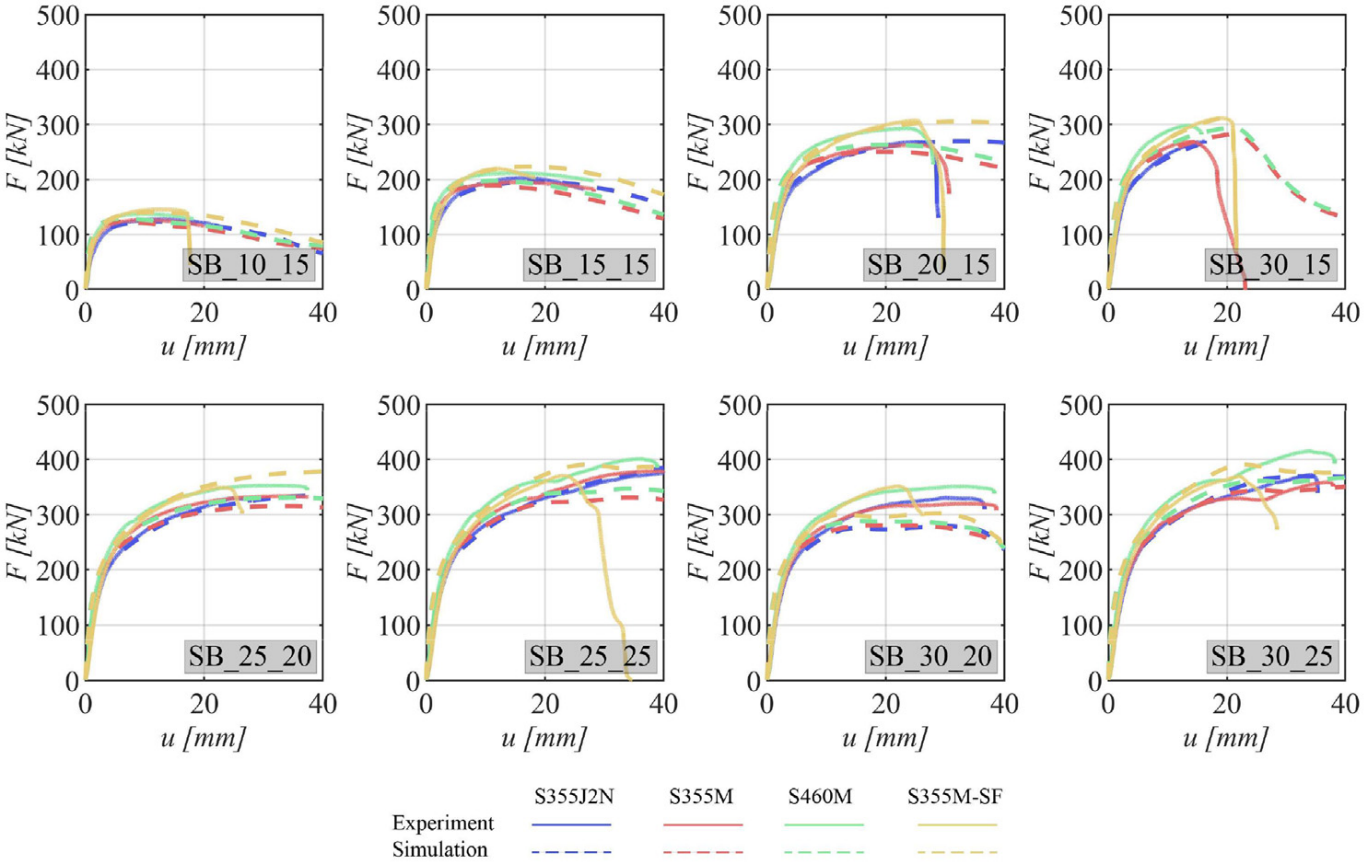
Load–deformation curves for specimens with one bolt. The experimental curve is shown as a continuous line and the curve obtained from the numerical simulation as dashed line. The steel grades are represented by the different colors.

**Fig. 8:**
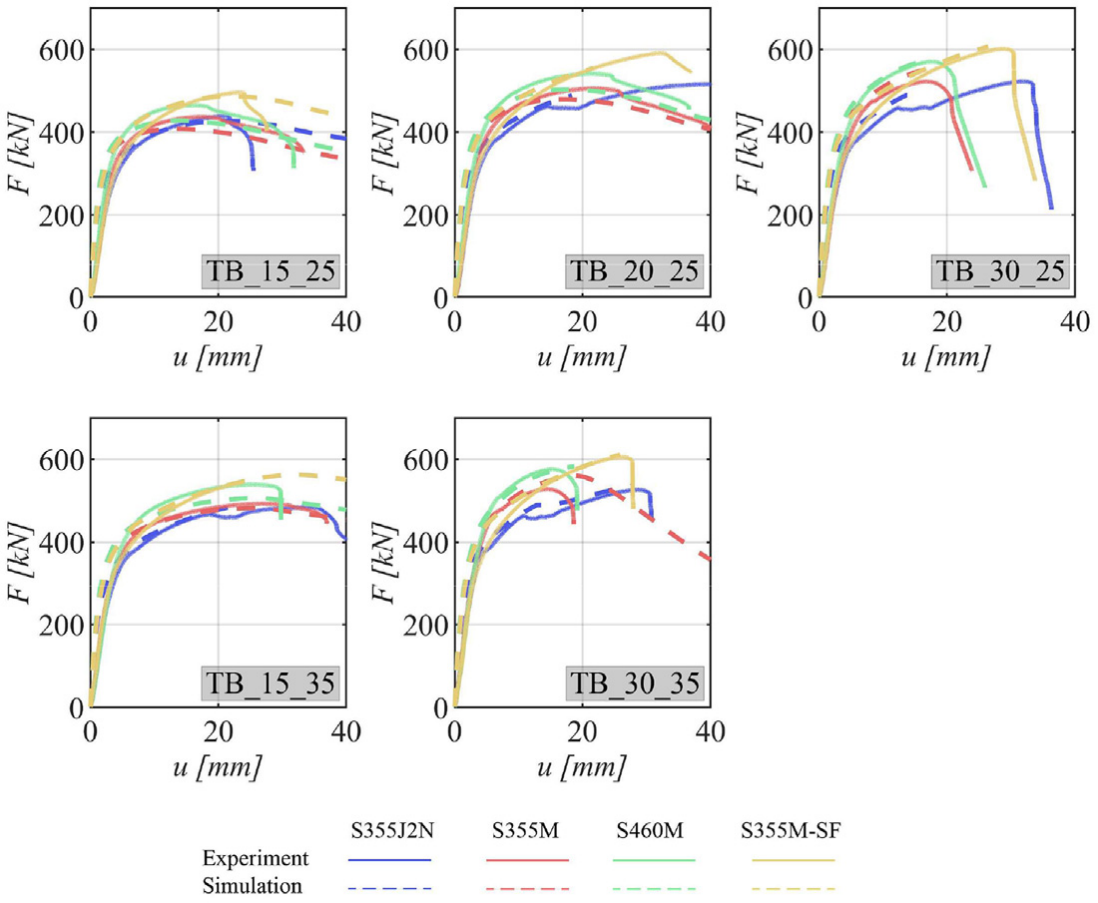
Load–deformation curves for specimens with two bolts. The experimental curve is shown as a continuous line and the curve obtained from the numerical simulation as dashed line. The steel grades are represented by the different colors.

**Fig. 9:**
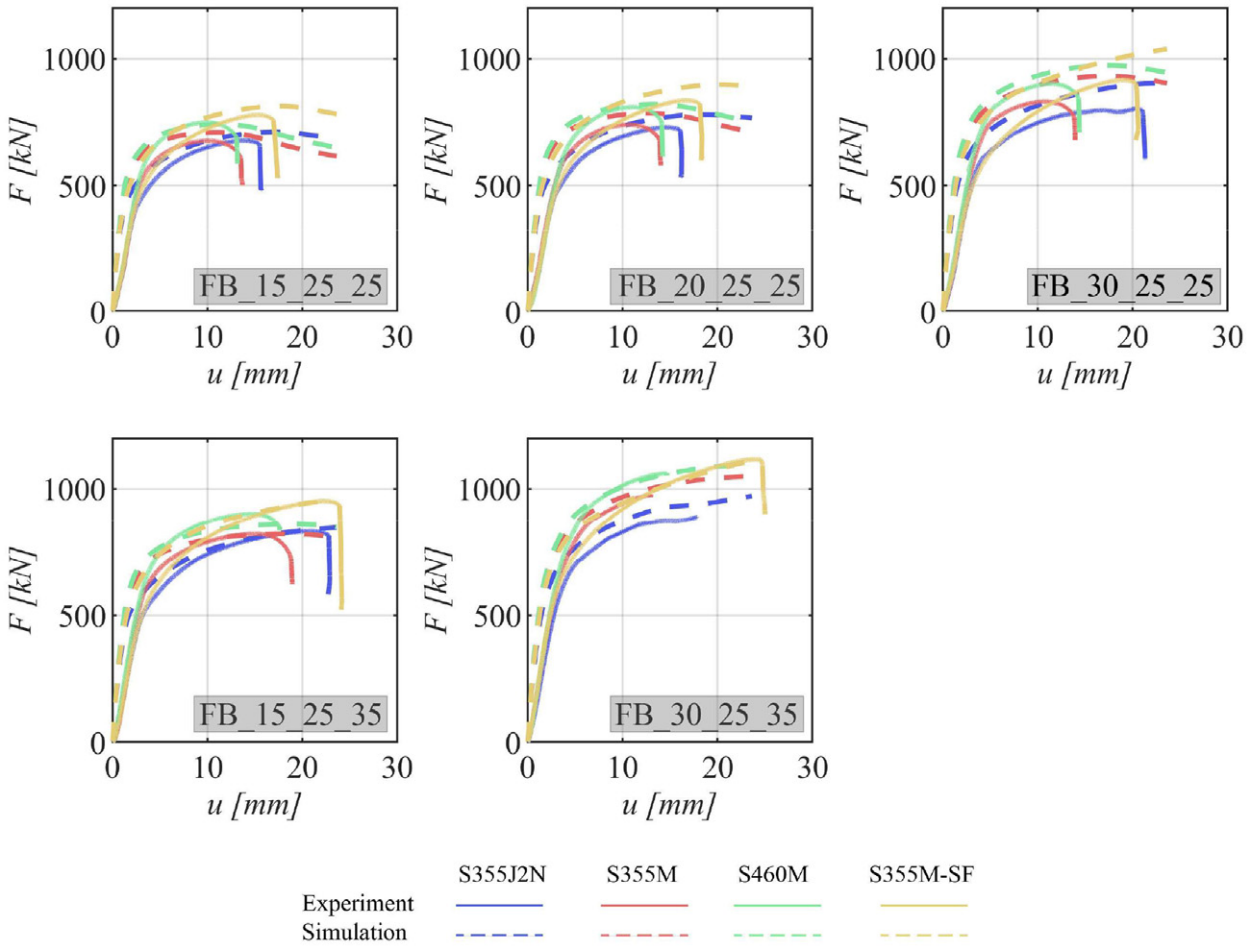
Load–deformation curves for specimens with four bolts. The experimental curve is shown as a continuous line and the curve obtained from the numerical simulation as dashed line. The steel grades are represented by the different colors.

The load-deformation curves for various specimen configurations are delineated in Fig. 7 for specimens featuring a single bolt, Fig. 8 for those with two bolts, and Fig. 9 for specimens equipped with four bolts. All these curves exhibit an initial steep linear region that rapidly transitions into the plastic deformation regime.

#### 3.3.7 Maximum reached force

**Fig. 10:**
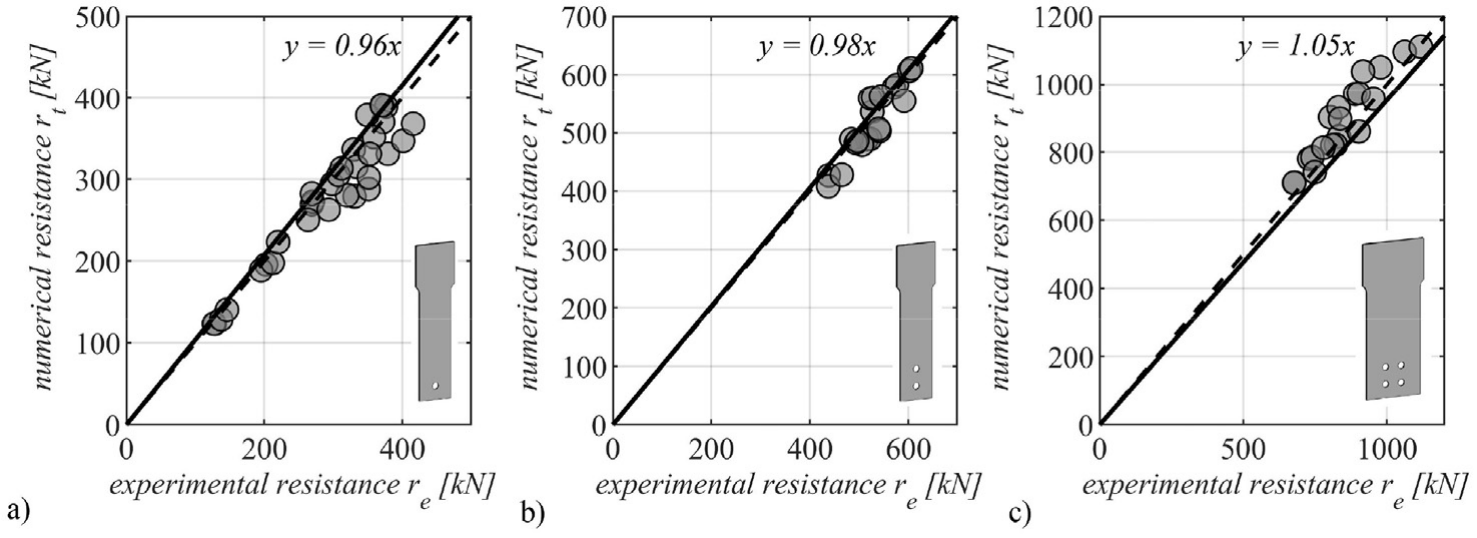
Comparison between experimental and numerical maximum resistance: a) for single bolt connections, b) for two bolts connections and c) for four bolts connections.

In this sub-section, we conduct a comparative analysis between the maximum force achieved in the experimental tests and the corresponding values derived from numerical simulations. This evaluation is presented separately for specimens with distinct bolt configurations: Fig. 10a for single-bolt specimens, Fig. 10b for specimens equipped with two bolts, and Fig. 10c for specimens featuring four bolts. The numerical resistance is graphed against the experimental resistance, and the dataset is subjected to linear regression analysis. The slope of the regression line is prominently displayed in the respective subfigure. A slope value less than 1.0 indicates that the numerical simulations predict forces of smaller magnitude than those observed in the experimental tests, and conversely, a slope exceeding 1.0 signifies the numerical simulations predict higher forces.

Overall, a high level of concordance between the results of experimental tests and numerical simulations is observed, particularly with respect to the maximum attained force. In the context of single-bolt connections, the numerical simulations tend to yield maximum force values that are generally smaller, exhibiting an average deviation of approximately 4%. This trend is consistent for connections involving two bolts, with an average deviation of merely 2%. In contrast, specimens featuring four bolts exhibit a contrary pattern, where the numerical simulations project higher maximum forces in comparison to the experimental tests, and the average deviation stands at 5%.

Nonetheless, it is imperative to recognize that the comparison between experiments and simulations is not solely contingent on the maximum achieved force; it also hinges on assessing the disparities in the load–deformation path across the entire duration of the test.

#### 3.3.8 Load–deformation path

**Fig. 11:**
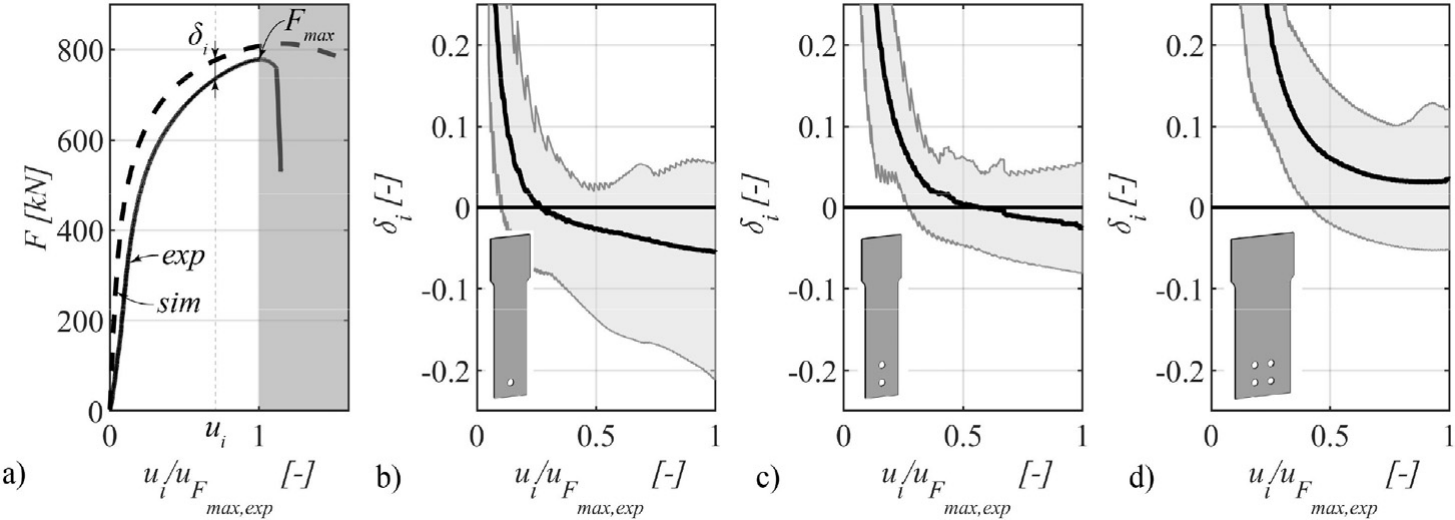
Difference in force between simulation and experiment versus normalized deformation until the deformation associated with the maximum force of the experiment is reached.

The load–deformation trajectory of the experimental tests is juxtaposed with that derived from the numerical simulations. To quantify this comparison, the load differential at a specific deformation point, denoted as *u_i_* is computed and subsequently normalized concerning the corresponding experimental load, as specified by Eq. (3). The outcome is a deviation expressed as a percentage in relation to the force observed in the experimental test. This methodology is exemplified using a representative specimen in Fig. 11a.

(3) $$\delta_{i}=\frac{F_{sim,u_{i}}-F_{\exp,u_{i}}}{F_{\exp,u_{i}}}$$

This comparative analysis is systematically applied across all specimen geometries and for each of the four steel grades subjected to investigation. Fig. 11 illustrates the divergence in load–deformation behavior between experimental tests and numerical simulations as a function of displacement. The displacement values are normalized in relation to the displacement associated with the maximum force recorded in the experimental tests, and the presentation commences from this specific point onward. For the sake of clarity, Fig. 11 is partitioned based on the number of bolts of the specimen. Accordingly, Fig. 11b pertains to specimens with one bolt, Fig. 11c corresponds to specimens with two bolts, and Fig. 11d is allocated to specimens featuring four bolts. The region where the test specimens are situated is demarcated in grey, and the collective mean value for tests sharing the same number of bolts is denoted by a black line.

As depicted in Fig. 11b, a noteworthy disparity in force emerges between the results of experimental tests and numerical simulations, particularly evident at small normalized deformations. Within this domain, the force computed via numerical simulations tends to exhibit higher values compared to the force derived from experimental tests. This discrepancy gradually diminishes with increasing normalized displacement, ultimately causing the mean value to transition into the negative domain. This signifies that the force predicted by the numerical simulations tends to be less than that obtained from experimental tests. Upon reaching the displacement corresponding to the maximum force observed in the experimental tests, the deviation manifests with a considerable spread, spanning from −20% to +5%, with an average deviation of −5.5%. In Fig. 11c, which pertains to specimens featuring two bolts, a reduced degree of scatter is generally observed. Following an initial substantial disparity observed at small normalized displacements, this disparity diminishes as normalized displacements increase and effectively approaches negligible levels when the deformation corresponding to the maximum force in the experimental tests is attained. At this juncture, an average deviation of −2.5% is notable. The discrepancy for specimens equipped with four bolts is elucidated in Fig. 11d, wherein a similar trend to the other two specimen types is generally observed. However, in contrast to the aforementioned cases, the mean deviation consistently resides in the positive domain, signifying that, on average, the numerical simulations consistently yield higher forces. As the displacement aligns with the point of maximum force in the experimental tests, the deviation exhibits substantial variability, spanning from −5% to +12%, with an average deviation of +3.5%.

### 3.4 Strains in the direction of force

**Fig. 12:**
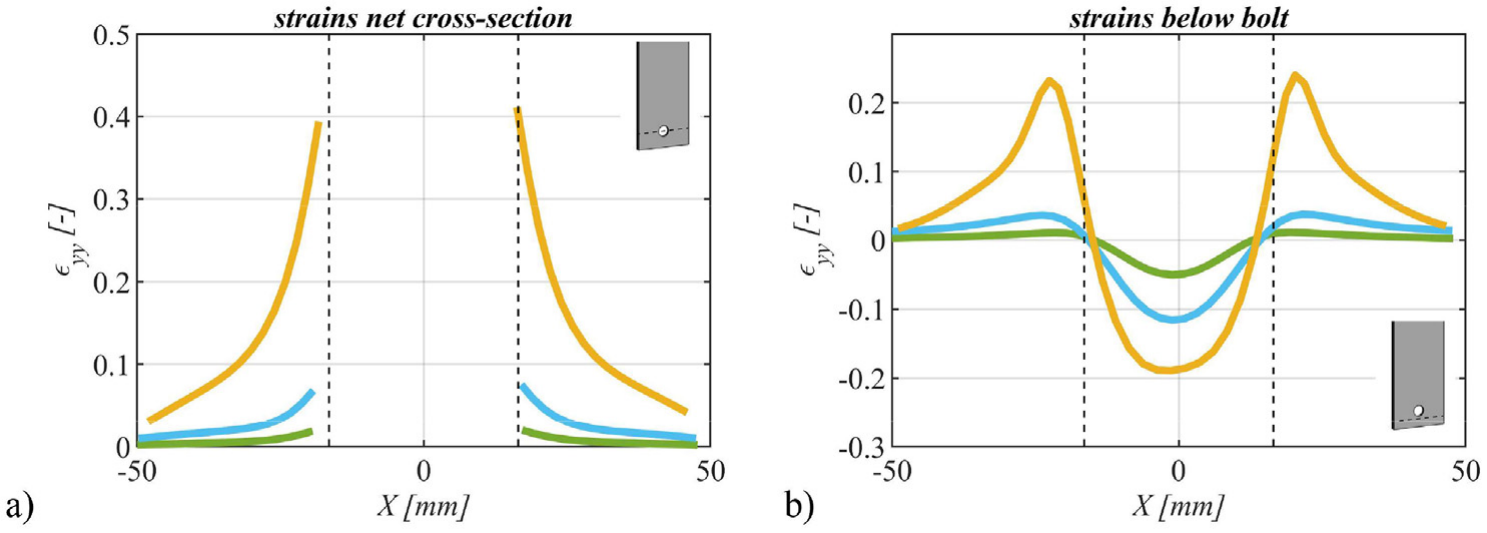
Strains in the direction of force for specimen SB_20_15 (S355M_SF) with one bolt plotted over the width of the specimen: a) in the net cross-section and b) below the bolt. Colors representing different force levels: green: 0.6F_u_, blue: 0.8F_u_ and orange: 1.0F_u_.

**Fig. 13:**
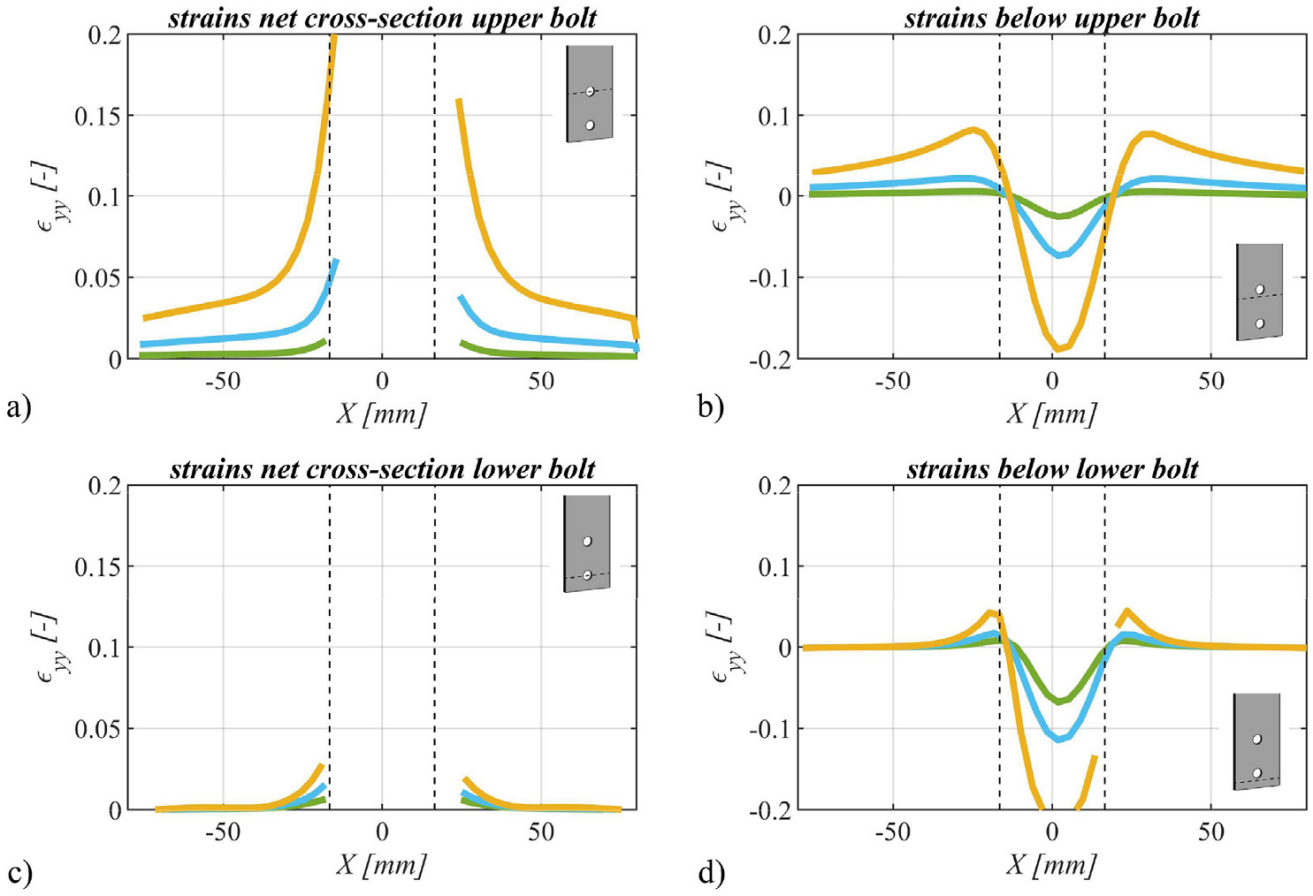
Strains in the direction of force for specimen TB_15_35 (S355M_SF) with two bolts plotted over the width of the specimen: a) in the net cross-section of the upper bolt, b) below the upper bolt, c) in the net cross-section of the lower bolt and d) below the lower bolt. Colors representing different force levels: green: 0.6F_u_, blue: 0.8F_u_ and orange: 1.0F_u_.

**Fig. 14:**
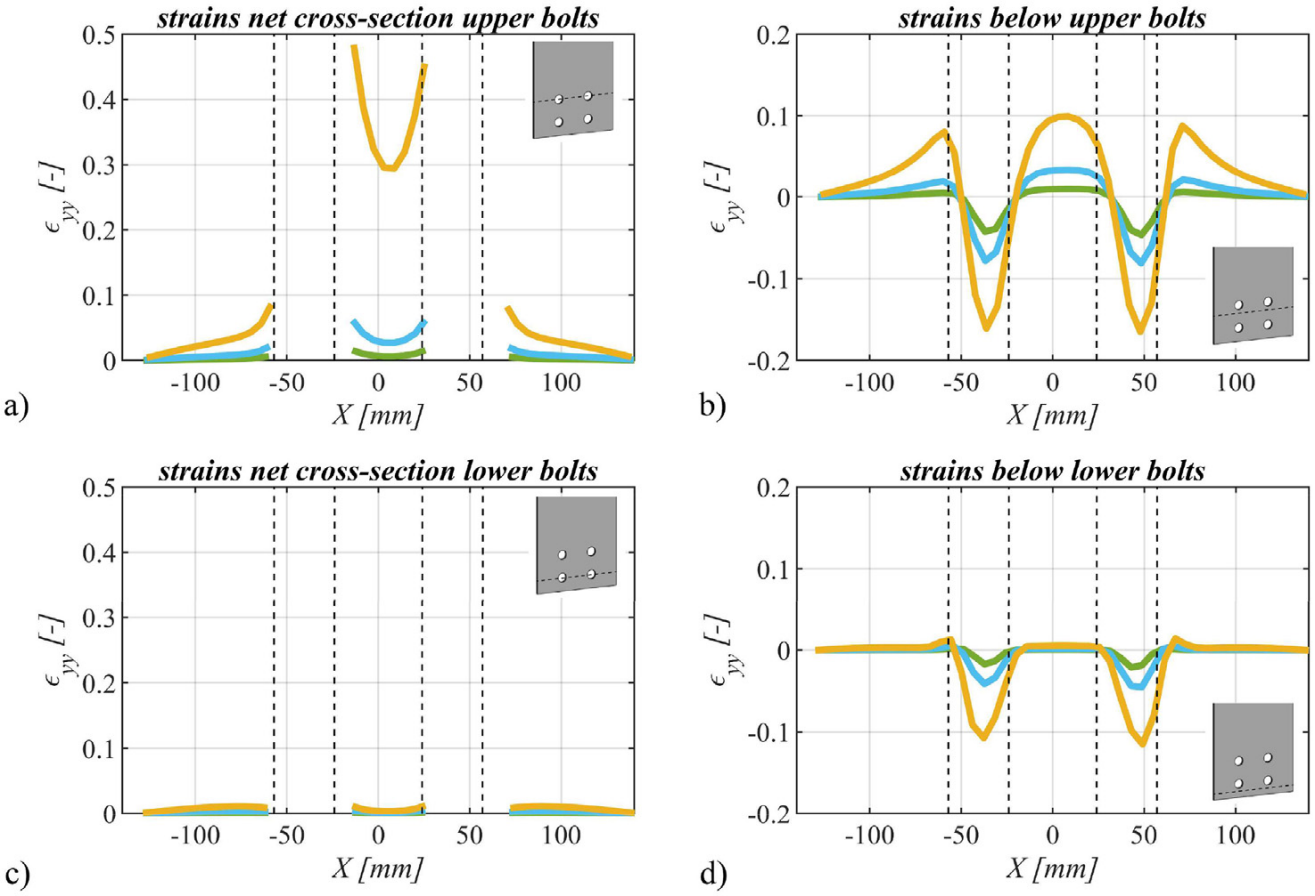
Strains in the direction of force for specimen FB_20_25_25 (S355M_SF) with four bolts plotted over the width of the specimen: a) in the net cross-section of the upper bolt, b) below the upper bolt, c) in the net cross-section of the lower bolt and d) below the lower bolt. Colors representing different force levels: green: 0.6F_u_, blue: 0.8F_u_ and orange: 1.0F_u_.

Utilizing the deformations recorded through the Digital Image Correlation (DIC) system, we computed strains in the direction of force. These strains were assessed across three distinct force magnitudes: i) 0.6⋅Fu, ii) 0.8⋅Fu, and iii) 1.0⋅Fu, thereby facilitating the observation of strain variations with increasing force. Exemplary representations of strains within the net cross-section(s) and beneath the bolt(s) are provided in Fig. 12 for a single bolt specimen, in Fig. 13 for a specimen with two bolts and in Fig. 14 for a four bolts specimen.

It is important to note that positively defined strains correspond to tensile strains, while negatively defined strains denote compressive strains. These diverse strain levels are distinguished by different colors, and the hole edges are schematically indicated using black dotted lines.

## Limitations

Not applicable.

## Ethics Statement

The authors confirm that they have read and follow the ethical requirements for publication in Data in Brief and confirm that the current work does not involve human subjects, animal experiments, or any data collected from social media platforms.

## CRediT authorship contribution statement

**Patrick Studer:** Conceptualization, Methodology, Validation, Formal analysis, Investigation, Writing – original draft, Project administration. **Andreas Taras:** Conceptualization, Writing – review & editing, Supervision, Funding acquisition.

## Data Availability

Dataset of hole bearing type connections of experimental tests and related numerical simulations (Original data) (ETH Research Collection).
